# Bone Immune Response to Materials, Part I: Titanium, PEEK and Copper in Comparison to Sham at 10 Days in Rabbit Tibia

**DOI:** 10.3390/jcm7120526

**Published:** 2018-12-07

**Authors:** Ricardo Trindade, Tomas Albrektsson, Silvia Galli, Zdenka Prgomet, Pentti Tengvall, Ann Wennerberg

**Affiliations:** 1 Department of Prosthodontics, Faculty of Odontology, The Sahlgrenska Academy, University of Gothenburg, 405 30 Gothenburg, Sweden; ann.wennerberg@odontologi.gu.se; 2 Department of Biomaterials, Institute of Clinical Sciences, University of Gothenburg, 405 30 Gothenburg, Sweden; tomas.albrektsson@biomaterials.gu.se (T.A.); pentti.tengvall@gu.se (P.T.); 3 Department of Prosthodontics, Faculty of Odontology, Malmö University, 205 06 Malmö, Sweden; silvia.galli@mau.se; 4 Department of Oral Pathology, Faculty of Odontology, Malmö University, 205 06 Malmö, Sweden; zdenka.prgomet@mau.se

**Keywords:** osseointegration, immune system, biomaterials, foreign body reaction, in vivo study

## Abstract

Bone anchored biomaterials have become an indispensable solution for the restoration of lost dental elements and for skeletal joint replacements. However, a thorough understanding is still lacking in terms of the biological mechanisms leading to osseointegration and its contrast, unwanted peri-implant bone loss. We have previously hypothesized on the participation of immune mechanisms in such processes, and later demonstrated enhanced bone immune activation up to 4 weeks around titanium implants. The current experimental study explored and compared in a rabbit tibia model after 10 days of healing time, the bone inflammation/immunological reaction at mRNA level towards titanium, polyether ether ketone (PEEK) and copper compared to a Sham control. Samples from the test and control sites were, after a healing period, processed for gene expression analysis (polymerase chain reaction, (qPCR)) and decalcified histology tissue analysis. All materials displayed immune activation and suppression of bone resorption, when compared to sham. The M1 (inflammatory)/M2 (reparative) -macrophage phenotype balance was correlated to the proximity and volume of bone growth at the implant vicinity, with titanium demonstrating a M2-phenotype at 10 days, whereas copper and PEEK were still dealing with a mixed M1- and M2-phenotype environment. Titanium was the only material showing adequate bone growth and proximity inside the implant threads. There was a consistent upregulation of (T-cell surface glycoprotein CD4) CD4 and downregulation of (T-cell transmembrane glycoprotein CD8) CD8, indicating a CD4-lymphocyte phenotype driven reaction around all materials at 10 days.

## 1. Introduction

Recent evidence suggests that biomaterials induce an immunomodulatory interaction with the host, and materials such as titanium or bone substitutes seem not at all inert upon contact with host bone [1]. The ultimate outcome of biomaterial implantation depends on the extent of the ensuing foreign body reaction (FBR) and related immune and inflammatory mechanisms; current scientific efforts are focusing on understanding this complex host reaction, in order to improve the behavior of implanted biomaterials [2]. However, the precise mechanisms of osseointegration are today not fully understood, especially the long-term immune recognition of implants.

The present authors have explored some immunological mechanisms in a previously published review [3], following the hypothesis that osseointegration is nothing but a special type of immune driven foreign body reaction to the implanted material, ending up in bone demarcation at or near the surface [4]. The main hypothesis was that implants are not biologically inert, meaning that the immune/inflammatory system, in this case with emphasis on the immune system, is activated when titanium interacts with host bone—A hypothesis that later was tested and verified in a recent 4 week experimental pilot animal study, where immunological markers representing macrophages, complement, neutrophils, lymphocytes and bone resorption markers were compared in osteotomy sites, with and without the presence of titanium implants [5]: Titanium sites, showed significant up-/or down-regulation of immune (and inflammatory) markers after 28 days, i.e., at a time point well into the bone remodeling phase The immune system was apparently activated through the complement system, displayed M1 (inflammatory) - and M2 (reparative) -macrophages phenotypes, neutrophil cytosolic factor 1 (NCF-1), and down regulation of markers related to osteoclastic activity. Comparatively, at an earlier stage (10 days) only the M2-macrophage (reparative) phenotype was identified around titanium, when compared to the sham site. From earlier studies immune complement is known to become activated at a very early time point around titanium, and materials are then recognized as foreign objects by inflammatory cells [6]. During bone healing, and after the acute inflammatory phase, macrophages and their classically described polarization into M1 (inflammatory) and M2 (reparative) phenotypes dominate, and are considered to be central in the host reaction to implanted biomaterials [7,8], but the precise in vivo mechanisms are still in need of a thorough clarification. Macrophages are also intimately related to bone biology, interacting closely with osteoblasts during bone formation (these macrophages are named Osteomacs), and also fusing into either osteoclasts or material related multinucleated giant cells (named Foreign Body Giant Cells), determining a further important role for macrophages when considering bone borne biomaterials [9].

In the earlier review [3], and pertaining the current manuscript, it was further hypothesized that the reason why different materials may or not achieve osseointegration is probably related, to some extent, to a persistent immune patrolling resulting in a modified inflammatory reaction around the different materials. These two concepts—That materials are not biologically inert and that a specific persistent immune-inflammatory balance or patrolling around different materials largely dictates whether osseointegration occurs or not—Are fundamental to our understanding of longer-term host reactions to materials in bone.

The aim of the present exploratory in vivo study is to test the hypothesis that different materials trigger different early immune/inflammatory responses upon implantation in rabbit bone, and that these different responses may be important for the establishment of osseointegration, or the ultimate lack of it.

## 2. Materials and Methods

The current study consists of an experiment in the rabbit proximal tibia (metaphysis), comparing bone healing on sites where osteotomies were performed and then either left to heal without the placement of a material (sham site- Sh), or had one of the three test materials placed for comparison: titanium (Ti), copper (Cu) or polyether ether ketone (PEEK). Each rabbit received one site of each group (two sites per tibia): Sh, Ti, Cu and PEEK. Ti and PEEK were placed on the right tibia and a Sham site was produced and Cu was introduced in the other osteotomy on the left tibia. All implants were machined with a turning process, with a threaded 0.6 mm pitch height, 3.75 mm width Branemark MkIII design. The Ti implants were made of commercially pure titanium grade IV. Implant manufacturers: Ti and PEEK implants were produced by Carlsson and Möller, Helsingborg, Sweden; Copper implants were produced by TL Medical Company, Molndal, Sweden.

The sham site also provokes an inflammatory reaction, which is still present at 10 days and is used as a baseline to compare with the immune reaction elicited by each of the different materials.

### 2.1. Surgical Procedure

This study was performed on 6 mature, female New Zealand White Rabbits (*n* = 6, weight 3 to 4 Kg), with the ethical approval from the Ethics Committee for Animal Research (number 13-011) of the École Nationale Vétérinaire D’Alfors, Maisons-Alfors, Val-de-Marne, France. All care was taken to minimize animal pain or discomfort during and after the surgical procedures. For the surgical procedures, the rabbits were placed under general anesthesia using a mixture of medetomidine (Domitor, Zoetis, Florham Park, NJ, USA), ketamine (Imalgène 1000, Merial, Lyon, France) and diazepam (Valium, Roche, Basel, Switzerland) for induction, then applying subcutaneous buprenorphine (Buprecare, Animalcare, York, UK) and intramuscular Meloxicam (Metacam; Boehringer Ingelheim Vetmedica, Inc., Ridgefield, CT, USA). A single incision was performed in the internal knee area on each side and the bone exposed for osteotomies and insertion of implants in the sites mentioned above. The surgical site was sutured with a resorbable suture (Vicryl 3-0, Ethicon, Cincinnati, OH, USA) and hemostasis achieved. Following surgery, Fentanyl patches (Duragesic, Janssen Pharmaceutica, Beerse, Belgium) were applied.

The osteotomies were produced with a sequence of increasing diameter twist drills, from 2 mm to 3.15 mm width, and a final countersink bur prepared the cortical part of the bone. The implants used were 3.75 mm in diameter.

The rabbits were housed in separate cages and were allowed to move and eat freely. At 10 days, the rabbits were sacrificed with a lethal injection of sodium pentobarbital (Euthasol, Virbac, Fort Worth, TX, USA). The 6 animals had the implants removed through unscrewing. On 4 of those animals, bone was collected with a 2 mm twist drill from the periphery of the Sh, Ti, Cu and PEEK sites on the most distal portion, and then processed for Gene Expression Analysis through quantitative polymerase chain reaction (qPCR). The 6 animals had the test sites then removed en bloc for histological processing.

### 2.2. Gene Expression Analysis—qPCR

The bone samples for gene expression analysis were collected from the distal side of the osteotomies of all four groups (following the removal of the implant from the implant sites), with a 2 mm twist drill that removed both cortical and marrow bone in the full length of the osteotomy, to enable the study of the 2 mm peri-implant bone area of each of the Sh, Ti, Cu and PEEK sites. The samples were immediately transferred to separate sterile plastic recipients containing RNA*later* medium (Ambion, Inc., Austin, TX, USA), for preservation. The samples were then refrigerated first at 4 °C and then stored at −20 °C until processing.

### 2.3. mRNA Isolation

Samples were homogenized using an ultrasound homogenizer (Sonoplus HD3100, Brandelin, Berlin, Germany) in 1 mL PureZOL and total RNA was isolated via column fractionalization using the Aurum^TM^ Total RNA Fatty and Fibrous Tissue Kit (Bio-Rad Laboratories Inc., Hercules, CA, USA) following the manufacturer’s instructions. All the samples were DNAse treated using an on-column DNAse I contained in the kit to remove genomic DNA. The RNA quantity for each sample was analyzed in the NanoDrop 2000 Spectrophotometer (Thermo Scientific, Wilmington, NC, USA). BioRad iScript cDNA synthesis kit (Bio-Rad Laboratories Inc., Hercules, CA, USA) was then used to convert mRNA into cDNA, following the manufacturer’s instructions.

qPCR primers (Tataa Biocenter, Gothenburg, Sweden) were designed following the National Center of Biotechnology Information (NCBI) Sequence database, including the local factors chosen in order to characterize the immune, inflammatory and bone metabolic pathways (Table 1 and Table 2). All primers had an efficiency between 90 and 110%.

### 2.4. Amplification Process

Five µL of SsoAdvanced SYBR^™^ Green Supermix (Bio-Rad Laboratories Inc., Hercules, CA, USA) and 1 µL of cDNA template together with 0.4 µM of forward and reverse primer were used in the qPCR reaction. Each cDNA sample was performed on duplicates. The thermal cycles were performed on the CFX Connect Real-Time System (Bio-Rad Laboratories Inc., Hercules, CA, USA). The CFX Manager Software 3.0 (Bio-Rad, Hercules, CA, USA) was used for the data analysis.

Three genes (GAPDH, ACT-β, LDHA) were selected as reference genes using the geNorm algorithm integrated in the CFX Manager Software. A quantification cycle (Cq) value of the chosen reference genes (Table 1 and Table 2) was used as control; hence the mean Cq value of each target gene (Table 1) was normalized against the reference gene’s Cq, giving the genes’ relative expression. For calculation of fold change, the ΔΔCq was used, comparing mRNA expressions from the different groups. Significance was set at *p* < 0.05 and the regulation threshold at ×2-fold change.

### 2.5. Decalcified Bone Histology

After removal of the implants from the studied Sh, Ti, Cu and PEEK sites on the 6 subjects, bone was removed en bloc and preserved in 10% formalin (4% buffered formaldehyde, VWR international, Leuven, Belgium) during 48 h for fixation. Samples were decalcified in ethylene diamine tetra-acetic acid (10% unbuffered EDTA; Milestone Srl, Sorisole, Italy) for 4 weeks, with weekly substitution of the EDTA solution, dehydrated and embedded in paraffin (Tissue-Tek TEC, Sakura Finetek Europe BV, Leiden, The Netherlands). Samples were sectioned (4 µm thick) with a microtome (Microm HM355S, Thermo Fischer Scientific, Walldorf, Germany) and stained with hematoxylin-eosin (HE) for histological analysis.

### 2.6. Statistical Analysis

The gene expression statistical analysis was performed using the *t* test built in the algorithm of the CFX Manager Software 3.0 package (BioRad, Hercules, CA, USA). The gene expression analysis was made pair wise, each material being evaluated against the Sham in each animal.

### 2.7. Surface Roughness

The surface roughness of each material was measured (following Wennerberg and Albrektsson guidelines (2000)) [10], with a white light 3D optical Profilometer, gbs, smart WLI extended (Gesellschaft für Bild und Signal verarbeitung mbH, Immenau, Germany) using a 50× objective. MountainsMap® Imaging Topography 7.4 (Digital Surf, Besancon, France) software was used to evaluate the data. Surface roughness parameters were calculated after removing errors of form and waviness. A gaussian filter with a size of 50 × 50 µm was used. The measuring area was 350 × 220 µm for all measurements, 3 copper, 3 titanium and 3 PEEK implants were measured, each implant on 9 sites (3 tops, 3 valleys and 3 flanks).

In order to characterize the surface in height, spatial and surface enlargement aspects 4 parameters were selected; S_a_ that describes the average height distribution measured in µm, S_ds_ which is a measure of the density of summits over the measured area, measured in 1/µm^2^, Ssk (skewness) a parameter that describes the asymmetry of the surface deviation from the mean plane and S_dr_ which describes the surface enlargement compared to a totally flat reference area, measured in %.

## 3. Results

### 3.1. Gene Expression Analysis

**Figure 1:**
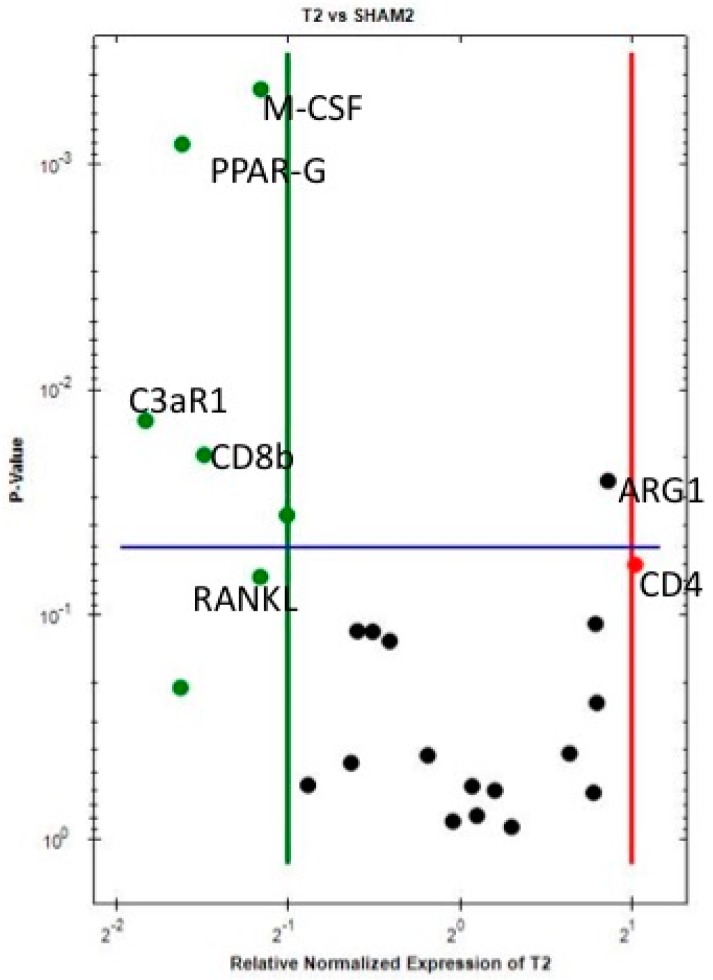
Volcano plot comparing the gene expression of Ti versus Sh at 10 days. Downregulation (vertical green line) and Upregulation (vertical red line) set a ×2 regulation. Statistical significance (horizontal blue line) set at *p* < 0.05—Marker is significant when above blue line.

Each material (Ti, Cu and PEEK) was compared against the Sh site for gene expression regarding the immunological reaction after 10 days of healing- and considering that the Sh site itself, also produces an immune-inflammatory reaction. The results show that when comparing Ti sites with Sh sites (Table 3 and Figure 1), ARG1 (M2-macrophage) is statistically significantly and almost 2-fold upregulated, while CD4 (T helper lymphocytes) is 2-fold upregulated and close to statistical significance. This indicates an activation of the immune system already at 10 days around Ti, when compared to Sh. On the other hand, the downregulation of both C3aR1 (complement component 3a receptor 1) and CD8 (T cytotoxic lymphocytes) was more than 2-fold and at a statistically significant level around Ti compared to Sh, which further supports the notion of an immunological involvement in the host response towards titanium, as it probably represents a biological feedback effect following activation of complement factor C3 and T cells at an earlier stage. Furthermore, peroxisome proliferator-activated receptor gamma (PPAR-γ) is significantly downregulated, while RANKL (Receptor activator of nuclear factor kappa-B ligand) and OPG (osteoprotegerin) showed a non-significant downregulation, indicating an environment around Ti where bone resorption is apparently suppressed. Macrophage colony-stimulating factor (M-CSF) is significantly downregulated, indicating suppression of cell (macrophage) fusion around Ti at 10 days, into either osteoclasts or foreign body giant cells (FBGCs).

**Figure 2:**
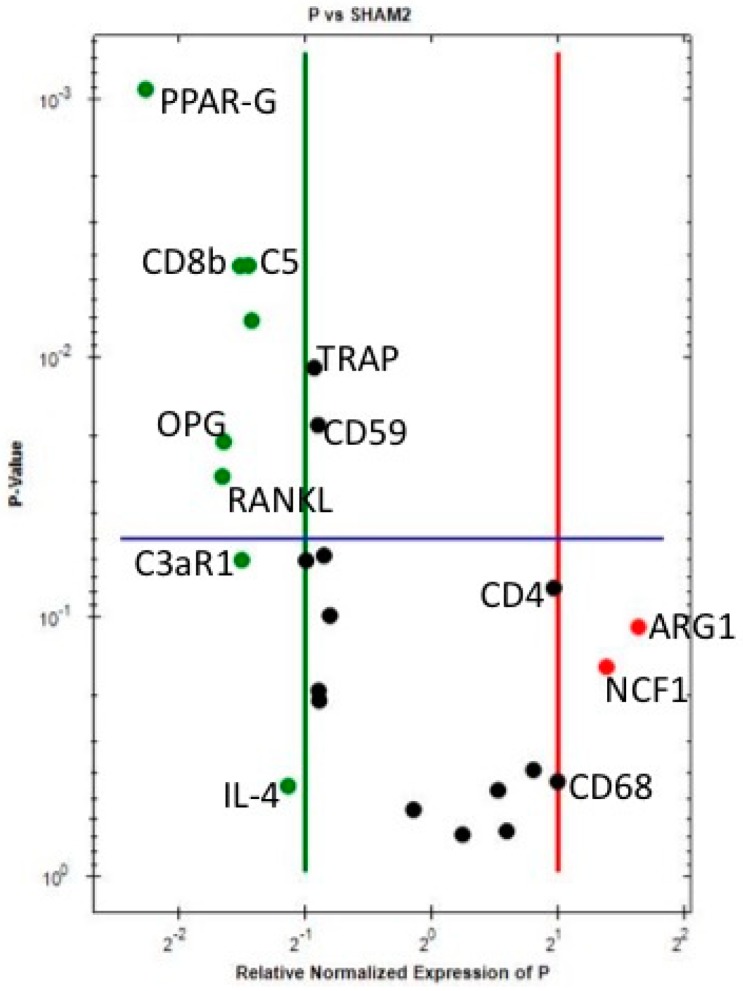
Volcano plot comparing the gene expression of PEEK versus Sh at 10 days. Downregulation (vertical green line) and Upregulation (vertical red line) set a ×2 regulation. Statistical significance (horizontal blue line) set at *p* < 0.05—Marker is significant when above blue line.

When comparing PEEK sites with Sh sites (Table 4 and Figure 2), even if not significantly, ARG1 (M2-macrophages), NCF-1 (neutrophils), CD68 (M1-macrophages) and CD4 (T helper cells) are upregulated 2-fold or more around PEEK when compared to Sh sites, indicating early immune activation around PEEK. Downregulation of CD8 (T cytotoxic cell—Significant), complement factors (C3aR1, CD55, CD59 and C5- the last two statistically significant), strongly adds to the notion of immune system involvement in the host reaction towards PEEK implants. The downregulation of PPAR-gamma, RANKL, OPG, TRAP (all statistically significant) and CATHK demonstrates the suppression of bone resorption around PEEK implants after 10 days of insertion in the bone.

**Figure 3:**
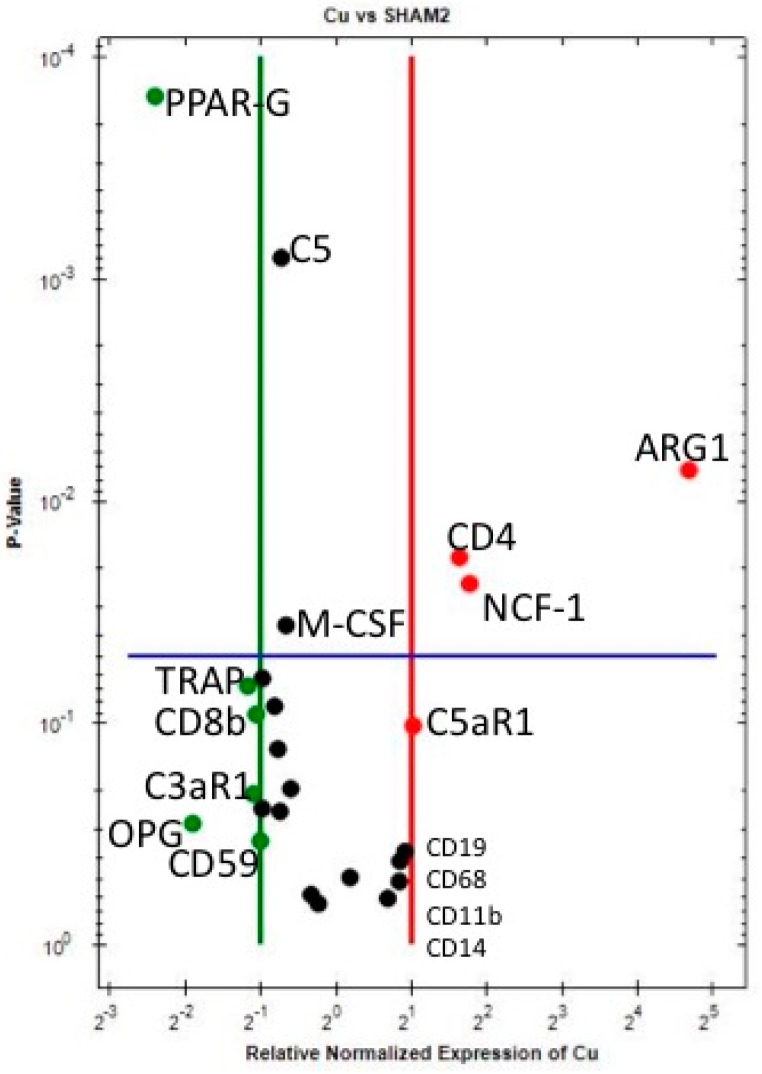
Volcano plot comparing the gene expression of Cu versus Sh at 10 days. Downregulation (vertical green line) and Upregulation (vertical red line) set a ×2 regulation. Statistical significance (horizontal blue line) set at *p* < 0.05—Marker is significant when above blue line.

Around Copper (Cu) implants, when compared to Sh (Table 5 and Figure 3) at 10 days, ARG1 (M2 macrophage), NCF1 (neutrophils) and CD4 (T helper cells) are statistically significantly upregulated. Furthermore, even if not reaching the statistical significance level of *p* < 0.05, CD19 (B cells), C5aR1 (complement C5 receptor 1), CD68, CD14 and CD11b (the latter three are M1-macrophage markers) are upregulated. These results demonstrate a strong immune activation around Cu upon contact with host bone tissue.

C5, CD59, CD55, CD46 and C3aR1 (complement factors) are downregulated, suggesting a feedback effect following complement activation (which is confirmed by C5aR1 upregulation).

CD8 (T cytotoxic cell) shows a tendency for downregulation around Cu.

The statistically significant downregulation of M-CSF, with the tendency for downregulation of IL-4, even if both not reaching a 2-fold change, suggests that at 10 days of implantation, macrophage fusion into either osteoclasts or FBGCs is suppressed around Cu—Similar to what was observed above around Ti. Additionally, as for Ti and PEEK, bone resorption is suppressed around Cu at 10 days, when compared to Sh, since PPAR-gamma is statistically significantly downregulated and the other bone resorption markers (RANKL, OPG, TRAP and CATHK) show a tendency for downregulation.

### 3.2. Decalcified Bone Histology

**Figure 4:**
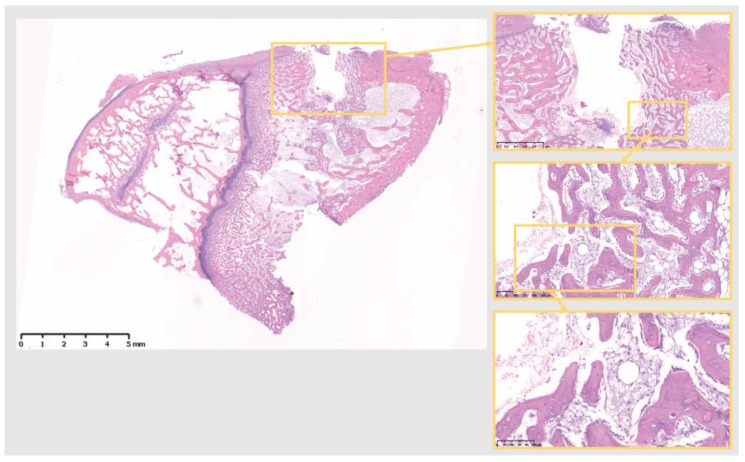
The 10 days Sh site. Bone remodeling with new bone formation around the osteotomy site. Defect is isolated from the marrow space. Scale bars, clockwise: 5 mm, 1 mm, 250 µm and 100 µm.

**Figure 5:**
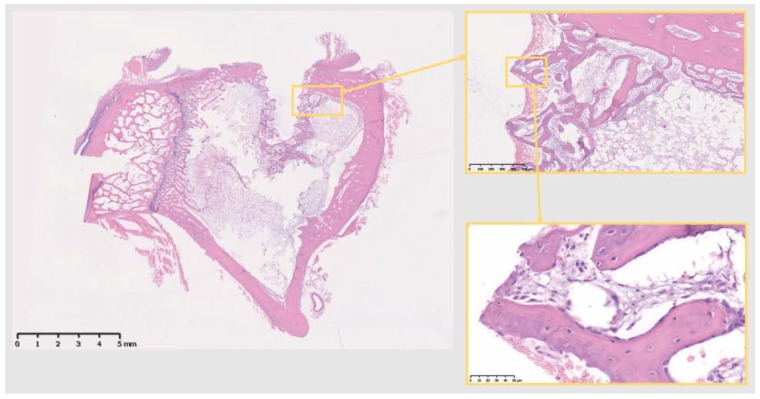
The 10 days Ti site. Bone remodeling and new bone formation around the implant site, isolating it from the marrow space. Scale bars, clockwise: 5 mm, 500 µm and 50 µm.

**Figure 6:**
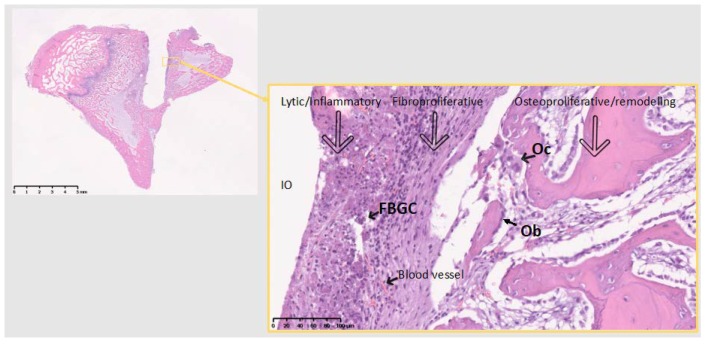
The 10 days Cu site. No bone on the immediate implant vicinity. FBGC, foreign body giant cells; Oc, osteoclast actively remodeling old bone; Ob, Seam of osteoblasts producing new bone (part of the remodeling); IO, Implant/Osteotomy. Reaction to Cu divided in 3 zones, representing the 3 phenomena around implant materials in the bone: From the implant surface Lytic/Inflammatory area, Fibroproliferative area and Osteoproliferative area. The latter two represent an attempt to isolate the material from the marrow cavity. No osseointegration is viable at this time point. Theoretically, around Ti the same phenomena exist, but at a different balance, allowing for osseointegration, through direct bon-to-implant contact. Inflammatory area is highly vascularized. Scale bars 5 mm (left) and 100 µm (right).

**Figure 7:**
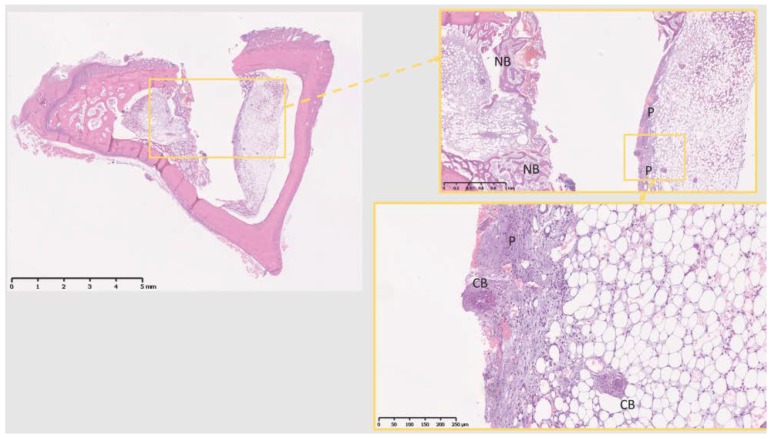
The 10 days PEEK. NB, new bone; forming only in the areas adjacent to cortical bone, while most other interfacial tissue shows no bone formation. P, proliferative area; no visible calcified tissue formation, but for some isolated calcified bone areas (CB, calcified bone). Scale bars, clockwise: 5 mm, 1 mm and 250 µm.

Decalcified histological sections of the four groups (Sham and the three different materials) were analyzed at a tissue level. The Sh sites display some bone formation around the osteotomy site, which is decreasing in size after 10 days, as the new bone fills in the osteotomy defect (Figure 4). Ti sites show new bone formation and bone remodeling in the thread areas (Figure 5). Cu sites display no bone formation at the interface of the implant site, showing a division (from the implant area) of a first layer of inflammatory cells with signs of cell lysis and some foreign body giant cells, followed by a proliferative area with parallel aligned fibers to the implant site, which in turn is followed outwards by an area of bone remodeling, more noticeable in areas closer to cortical bone, where osteoblasts and osteoclasts can be observed remodeling the old bone (Figure 6). PEEK sites present very little new bone formation/remodeling areas close to the implant interface, confined to areas in cortical bone proximity, whereas most of the interface presents only a thin proliferative area parallel to the implant site, mostly consisting of fibrous tissue and with very few calcified islands (Figure 7). The cellular components have all been clearly identified, neutrophils, macrophages, osteoclasts, osteoblasts, and also foreign body giant cells. However, quantification has not been performed, as it is not within the scope of the present study.

### 3.3. Surface Roughness

Table 6 shows the results on each material surface roughness analysis. The surface enlargement, which is depending on both height and the density of the surface irregularities, was smallest for the copper implants mostly depending on the lower height deviation compared to titanium and PEEK. Titanium and PEEK had a frequency distribution close to zero, while copper implants had slightly more peaks than pits. In terms of height deviation, titanium demonstrated the roughest surface.

## 4. Discussion

The present results demonstrate the immune system activation around Ti, PEEK and Cu once in contact with host bone, after 10 days of implantation- this demonstrates that, eventually, all materials render an immune activation, when in contact with bone.

Macrophage polarization, between M1-macrophage and M2-macrophage phenotypes, has been highlighted as a determining factor in the foreign body reaction, i.e., how host tissues interact with biomaterials [7,8]. M1-macrophages present a pro-inflammatory phenotype, while M2-macrophages have been identified as anti-inflammatory cells, participating in wound healing—Namely in the healing phase of acute inflammation—And also in chronic inflammation associated with immunological diseases, such as Rheumatoid Arthritis and Psoriasis [11]. M1-macrophages are described as induced by interferon-gamma (IFN-γ), while M2 macrophages are described as induced by e.g., IL-4 and IL-13 [8].

One of the main findings in this study is the importance of the M1/M2-macrophage (M1/M2) immunological balance in osseointegration already at this early stage: Titanium displays a reparative/anti-inflammatory M2-macrophage (M2) phenotype (ARG1), whereas Cu and PEEK are still dealing with a mixed pro-inflammatory M1-macrophage (M1) and M2 anti-inflammatory type of reaction (CD68, CD14 and CD11b; ARG1, respectively). The early preferential polarization towards a M2 phenotype around Ti, in the M1/M2 balance, probably explains the event of osseointegration being successful around Ti and not around the other materials—A fact already hypothesized in our previous work [3] and further discussed below. 

Another important, and unpredicted, finding is the tilting towards the CD4 T-cell phenotype and suppression of the CD8 T-cell phenotype around all materials, opening a window to further understanding the immune reaction to biomaterials in the bone, by demonstrating the participation of T-cells and indicating an early specific T-cell phenotype (discussed below).

The current results confirm the present authors’ previously published results comparing Ti and Sh immune responses, where immune activation around Ti implants was demonstrated in a femur study at 10 days and even more so at 28 days after implantation, i.e., outside the major inflammatory period [12].

For the present experiment, Ti was chosen as an already studied material, and the results of the previous study were confirmed, while PEEK was chosen for its perceived bio-inertness [13] and Cu for its known induction of a stronger inflammatory reaction when in contact with tissues—As demonstrated by Suska et al. 2008 in a rat soft tissue model [14]. As already mentioned, Ti displays mostly a reparative type-2 phenotype (ARG1 and CD4), whereas around Cu (ARG1, NCF1, CD11b, CD68, CD14, C5aR1, CD19) and PEEK (ARG1, CD68, NCF1, CD4) there is still a mixed environment, with pro-inflammatory and anti-inflammatory/reparative elements, which may explain the different bone tissue reaction towards the different materials at a tissue level (supported by the histological findings in this study). Even if some of these markers are not expressed with a statistically significant difference in value, most pass the ×2 threshold in regulation fold-change or are very close to that value; hence, their interpretation is crucial to understand the biological events and the osteoimmunology in relation to the studied materials.

The three materials have shown up-regulation of ARG1, indicating a reparative type-2 anti-inflammatory reaction (M2- macrophages and ILC-2). PEEK and Cu also show a M1-macrophage pro-inflammatory type of reaction, meaning that at 10 days the host tissue is not yet tilting the balance towards a full reparative mode around Cu and PEEK, which may explain, at least in part, the results at tissue level. Hence, the present results confirm the notion of macrophages being central in host reaction to biomaterials, with a decisive role already at 10 days- it would be interesting to study how the biological immune process develops at a later time point, whether it resolves, maintains or increases around Cu and PEEK.

Furthermore, the results show activation of CD4+ T-cells around all materials at 10 days, whereas the CD8+ T-cell phenotype is suppressed. These findings demonstrate the participation of T-cells in the bone healing process around solid biomaterials, although it is not known whether solely an innate or also an adaptive type of immune reaction is present—Classically, the host reaction to biomaterials is perceived as an innate immunological process [5], hence indicating T-cell activity through cytokines, rather than an antigen-antibody interaction. Furthermore, ARG1, which shows a tendency for upregulation in the three materials compared to Sh, is also expressed by type-2 innate lymphoid-cells (ILC-2) [15], supporting an innate immune mechanism. T-helper cells (CD4+) are involved in the regulation of immune responses at many levels, such as interaction with macrophages and the recruitment of neutrophils [16]. Regulatory T-cells (Treg) are also CD4+, and are responsible for suppressing immune inflammatory responses to allow reparative processes, being important, for instance, in halting some forms of autoimmune diseases [17]; hence, upregulation of CD4 most probably indicates an immunologically driven reaction towards tissue repair and proliferation around the studied materials, which has also been suggested by other authors [18].

The statistically significant upregulation of NCF1 around Cu and a similar upregulation around PEEK (even if not statistically significant) already at 10 days, highlights the role of neutrophils in the host-biomaterial interaction; however, in our previous study [5], Ti showed a statistically significant upregulation of NCF1 only after 28 days of healing, which implies a stronger inflammatory reaction around Cu and PEEK at an early stage that may further help dictate a soft tissue formation around these materials, not enabling bone deposition at the implant surface. At an earlier stage in the healing response, neutrophils participate in the inflammatory reaction, although changing phenotype at a later stage in this response, which may be the result of either macrophages inhibiting neutrophil apoptosis for continued neutrophil local performance, or the possible participation in the reparative process, mainly through an enhancing effect on vascularization [19,20]. Vascularization is of particular importance for the early development of tissue around the implants in an attempt to isolate these from the marrow compartment during the foreign body reaction; this is especially important considering that bone is a hypoxic tissue [21].

Complement factors seem mostly suppressed around all of the materials studied, at 10 days. The complement system, however, is complex and self-regulated [22]. The results show that the complement components are mostly downregulated, probably reflecting an inhibitory reaction to an earlier complement activation during the initial healing phase- thus indicating the possible involvement of the complement system from an early time point in the host reaction to implanted materials in the bone.

Furthermore, bone resorption markers were downregulated around all three materials at 10 days, when compared to Sh sites. This demonstrates a bone resorption suppression in the immediate implant environment from an early stage, when compared to our previous study, where this was mostly perceived at a later time point (28 days) around Ti [5]. Hence, a bone forming environment is already being developed around materials from an early healing stage and within the inflammatory period.

Further studies including protein identification techniques are recommended to confirm the gene expression outcomes presented here and rule out possible post-transcriptional or post-translational changes in the biological response.

Copper presents with extensive apoptosis around the surface; one would attribute this to the toxicity of Copper ions, but the surface topography may play an important role in determining the phenotype of Macrophages here: Copper compared to Sham shows an upregulation of ARG1, indicating M2-macrophages already at 10 days (25 times up regulated in Cu, while Ti and PEEK show a less pronounced increase in ARG1, even if also relevant). A possible conclusion is that the chemical aspect of Copper surface is likely a major factor, given the apoptosis seen, but that the surface topography also has to be considered, given that current evidence on M1/M2-macrophage polarization has a clear link to surface topography [8], which relates to our results both on surface analysis and gene expression analysis. Further studies are hence necessary to understand the role of both surface topography and chemistry, which likely differs between different materials. The surface may play a role in the macrophage phenotype, however, in the present study, Cu did not demonstrate an exceptional roughness, rather similar to commercial Ti implants produced with grade 4 Ti. Therefore, the topography may have had an influence but not likely a major one. Recent in vitro studies have shown the effect of surface chemistry and topography in macrophage polarization. Zhang H et al. have demonstrated the surface chemistry immunomodulatory effect of amine silanized titanium, which reduced inflammation and promoted M2 polarization of macrophages [23], while Gao L et al. have demonstrated a M1- to M2-macrophage switch, through surface release of IL-4 [24]. Regarding surface topography, Shayan M et al. have demonstrated both in vitro and in a soft tissue in vivo study, that implant surface nanopatterning is able to selectively polarize macrophages towards M2, hence modulating the immune response to the selected biomaterial [25]. These studies concur with the current bone tissue in vivo experimental results, which demonstrated that different materials modulate the host immune response through the polarization of macrophages, where Ti promotes an early shift to a M2-macrophage phenotype- with the inherent consequences observed at the tissue level, and which may explain the clinical osseointegration seen around Ti implants in bone.

Decalcified histology of specimens from the four groups shows that only Ti develops a structured thread infill of new bone, at 10 days. All the groups form an area of several cell layers clearly isolating the implant material (or osteotomy site in Sh), although Cu and PEEK fail to produce an adequate volume of osseous tissue, showing mostly soft tissue in the interfacial zone. In fact, the present histological results support the published work by Osborne and Newesley (1980), indicating contact osteogenesis around “well tolerated” biomaterials and distance osteogenesis around “less well tolerated” biomaterials [26]. This difference can be explained by the above gene expression analysis, where Ti shows a more reparative environment at such an early stage, when compared to PEEK and Cu.

## 5. Conclusions/Summary

1. All three materials display immune/inflammatory system activation at 10 days;
2. A more favourable macrophage M1/M2 balance likely leads to a better osseointegration of Ti, as compared to Cu and PEEK:
3. A clearer M2 anti-inflammatory/reparative regulation around Ti at 10 days;
4. A mixture of M1 and M2 (pro- and anti-inflammatory, respectively) regulation around Cu and PEEK (more pronounced around Cu);
5. T-lymphocytes participate in the foreign body reaction to biomaterials at an early stage;
6. T-cells may act through a CD4+ phenotype (T_helper_/T_reg_), suppressing the CD8+ T_cytotoxic_ type of reaction at 10 days;
7. The up-regulation of the neutrophil specific factor NCF-1 around Cu and PEEK, indicates a higher inflammatory activity and may in part contribute to an inferior osseointegration on materials other than Ti;
8. Complement system seems predominantly downregulated around materials at 10 days, when compared to the Sh;
9. Bone forming environment (suppression of bone resorption) develops around all three implanted materials at an early stage, and within the inflammatory period;
10. At tissue level, only Ti seems to lead to osseointegration; PEEK and Cu show little or no implant related bone formation (respectively)—Which probably reflects the slightly more pronounced immune activation around the latter materials at this early stage;
11. Surface topography may play a role in macrophage phenotype and on the ultimate tissue level reaction to biomaterials, but further studies are needed.

The present results indicate that Ti osseointegration likely arises from a material-specific inflammatory/immune process leading to a shorter pro-inflammatory period and earlier reparative process, starting still within the inflammatory period and promoting bone apposition on Ti implant surfaces. It is further confirmed that all materials trigger an immune activation, even by materials like PEEK, previously considered as bio-inert. Different materials thus display different inflammatory balances in their vicinity, partly controlled by the immune system. Longer-term studies are necessary to better comprehend the immunobiology and tissue performance beyond the inflammatory period around established and new biomaterials.

## Figures and Tables

**Table 1:** Gene sequences.

Primer	Forward Sequence	Reverse Sequence	Accession nr/Transcript ID
**NCF-1**	TTCATCCGCCACATTGCCC	GTCCTGCCACTTCACCAAGA	NM_001082102.1
**CD68**	TTTCCCCAGCTCTCCACCTC	CGATGATGAGGGGCACCAAG	ENSOCUT00000010382
**CD11b**	TTCAACCTGGAGACTGAGAACAC	TCAAACTGGACCACGCTCTG	ENSOCUT00000001589
**CD14**	TCTGAAAATCCTGGGCTGGG	TTCATTCCCGCGTTCCGTAG	ENSOCUT00000004218
**ARG1**	GGATCATTGGAGCCCCTTTCTC	TCAAGCAGACCAGCCTTTCTC	NM_001082108.1
**IL-4**	CTACCTCCACCACAAGGTGTC	CCAGTGTAGTCTGTCTGGCTT	ENSOCUT00000024099
**IL-13**	GCAGCCTCGTATCCCCAG	GGTTGACGCTCCACACCA	ENSOCUT00000000154
**M-CSF**	GGAACTCTCGCTCAGGCTC	ACATTCTTGATCTTCTCCAGCAAC	ENSOCUT00000030714
**OPG**	TGTGTGAATGCGAGGAAGGG	AACTGTATTCCGCTCTGGGG	ENSOCUT00000011149
**RANKL**	GAAGGTTCATGGTTCGATCTGG	CCAAGAGGACAGGCTCACTTT	ENSOCUT00000024354
**TRAP**	TTACTTCAGTGGCGTGCAGA	CGATCTGGGCTGAGACGTTG	NM_001081988.1
**CathK**	GGAACCGGGGCATTGACTCT	TGTACCCTCTGCATTTGGCTG	NM_001082641.1
**PPAR-** **γ**	CAAGGCGAGGGCGATCTT	ATGCGGATGGCGACTTCTTT	NM_001082148.1
**C3**	ACTCTGTCGAGAAGGAACGGG	CCTTGATTTGTTGATGCTGGCTG	NM_001082286.1
**C3aR1**	CATGTCAGTCAACCCCTGCT	GCGAATGGTTTTGCTCCCTG	ENSOCUT00000007435
**CD46**	TCCTGCTGTTCACTTTCTCGG	CATGTTCCCATCCTTGTTTACACTT	ENSOCUT00000033915
**CD55**	TGGTGTTGGGTGGAGTGACC	AGAGTGAAGCCTCTGTTGCATT	ENSOCUT00000031985
**CD59**	ACCACTGTCTCCTCCCAAGT	GCAATCTTCATACCGCCAACA	NM_001082712.1
**C5**	TCCAAAACTCTGCAACCTTAACA	AAATGCTTTGACACAACTTCCA	ENSOCUT00000005683
**C5aR1**	ACGTCAACTGCTGCATCAACC	AGGCTGGGGAGAGACTTGC	ENSOCUT00000029180
**CD3**	CCTGGGGACAGGAAGATGATGAC	CAGCACCACACGGGTTCCA	NM_001082001.1
**CD4**	CAACTGGAAACATGCGAACCA	TTGATGACCAGGGGGAAAGA	NM_001082313.2
**CD8**	GGCGTCTACTTCTGCATGACC	GAACCGGCACACTCTCTTCT	ENSOCUT00000009383
**CD19**	GGATGTATGTCTGTCGCCGT	AAGCAAAGCCACAACTGGAA	ENSOCUT00000028895
**GAPDH**	GGTGAAGGTCGGAGTGAACGG	CATGTAGACCATGTAGTGGAGGTCA	NM_001082253.1
**ACT-β**	TCATTCCAAATATCGTGAGATGCC	TACACAAATGCGATGCTGCC	NM_001101683.1
**LDHA**	TGCAGACAAGGAACAGTGGA	CCCAGGTAGTGTAGCCCTT	NM_001082277.1

NCF-1, neutrophil cytosolic factor 1; CD68, macrosialin; CD11b, macrophage marker; CD14, monocyte differentiation antigen CD14; ARG1, Arginase 1; IL-4, Interleukin 4; IL-13, Interleukin 13; M-CSF, colony stimulating factor-macrophage; OPG, osteoprotegerin; RANKL, Receptor activator of nuclear factor kappa-B ligand; TRAP, tartrate resistant acid phosphatase; CathK, cathepsin K; PPAR-γ, peroxisome proliferator activated receptor gamma; C3, complement component 3; C3aR1, complement component 3a receptor 1; CD46, complement regulatory protein; CD55, decay accelerating factor for complement; CD59, complement regulatory protein; C5, complement component 5; C5aR1, complement component 5a receptor 1; CD3, T-cell surface glycoprotein CD3; CD4, T-cell surface glycoprotein CD4; CD8, T-cell transmembrane glycoprotein CD8; CD19, B-lymphocyte surface protein CD19; GAPDH, glyceraldehyde-3-phosphate dehydrogenase; ACT-β, actin beta; LDHA, lactate dehydrogenase A.

**Table 2:** Correspondence between studied gene expression and biological entities.

Biological Entity	Gene
Neutrophil	NCF-1
Macrophage	CD68, CD11b, CD14, ARG1
Macrophage fusion	IL-4, IL-13, M-CSF
Bone resorption	OPG, RANKL, TRAP, CathK, PPAR-γ
Complement	Activation: C3, C3aR1, C5, C5aR1 Inhibition: CD46, CD55, CD59
T-lymphocytes	CD3, CD4, CD8
B-lymphocytes	CD19
Reference genes	GAPDH, ACT-β, LDHA

**Table 3:** Gene expression Ti vs. Sham.

**Marker**	**Down-Regulation**	***p*-Value**
ARG1	1.82	0.0254
CD4	2.03	0.0598
**Marker**	**Down-Regulation**	***p*-Value**
M-CSF	−2.23	0.0004
PPAR-G	−3.07	0.0008
RANKL	−2.24	0.0678
OPG	−1.85	0.5711
C3aR1	−3.55	0.0137
CD8b	−2.80	0.0195

**Table 4:** Gene expression PEEK vs. Sham.

**Marker**	**Down-Regulation**	***p*-Value**
ARG1	3.11	0.1091
CD68	2.00	0.4304
NCF1	2.61	0.1556
CD4	1.95	0.0771
**Marker**	**Down-Regulation**	***p*-Value**
PPAR-G	−4.81	0.0009
RANKL	−3.16	0.0286
OPG	−3.13	0.0210
TRAP	−1.91	0.0109
CATHK	−1.75	0.0985
C5	−2.73	0.0044
CD59	−1.87	0.0181
CD55	−1.81	0.0578
C3aR1	−2.84	0.0601
CD8b	−2.86	0.0044

**Table 5:** Gene expression Cu vs. Sham.

**Marker**	**Up-Regulation**	***p*-Value**
ARG1	25.74	0.0072
NCF1	3.41	0.0234
CD4	3.11	0.0178
CD19	1.88	0.3768
C5aR1	2.03	0.1021
CD68	1.80	0.4153
CD11b	1.79	0.5131
CD14	1.61	0.6120
**Marker**	**Down-Regulation**	***p*-Value**
PPAR-G	−5.28	0.0001
RANKL	−1.71	0.1301
OPG	−3.74	0.2815
TRAP	−2.25	0.0676
C5	−1.66	0.0007
CD59	−2.01	0.3376
C3aR1	−2.14	0.2071
CD8b	−2.09	0.0906

**Table 6:** Surface roughness analysis.

Surface Roughness	S_a_ µm Mean	S_sk_ Mean	S_ds_ 1/µm^2^ Mean	S_dr_ % Mean
Copper	0.40	1.2	0.28	47
Titanium	0.75	−0.02	0.25	66
PEEK	0.56	−0.23	0.31	69

S_a_, describes the average height distribution measured in µm; S_ds_, a measure of the density of summits over the measured area, measured in 1/µm^2^; S_sk_ (skewness), a parameter that describes the asymmetry of the surface deviation from the mean plane; S_dr_, describes the surface enlargement compared to a totally flat reference area, measured in %.
